# Supplementary material for: Conformational landscapes of rigid and flexible molecules explored with variable temperature ion mobility-mass spectrometry
Source: Nat Commun. 2025 May 6;16:4183. doi: 10.1038/s41467-025-59065-x (PMC12052783; doi:10.1038/s41467-025-59065-x)
Supplement: Supplementary file 1 — Supplementary Information [file 41467_2025_59065_MOESM1_ESM.pdf]

Supplementary Information

Conformational landscapes of rigid and flexible molecules explored with variable temperature ion mobility-mass spectrometry

Xudong Wang[1], Emma Norgate[1], Junxiao Dai[1], Florian Benoit[1], Tony Bristow[2], Richard M. England[3], Jason Kalapothakis[1] and Perdita E. Barran[1,*]

[1]Michael Barber Centre for Collaborative Mass Spectrometry, Manchester Institute of Biotechnology, Department of Chemistry, The University of Manchester, 131 Princess Street, Manchester, M1 7DN, UK. [2]Chemical Development, Pharmaceutical Technology and Development, Operations, AstraZeneca, Charter Way, Macclesfield, SK102NA, UK. [3]Advanced Drug Delivery, Pharmaceutical Sciences, R&D, AstraZeneca, Macclesfield, UK SK10 2NA.

[*]Corresponding Author: perdita.barran@manchester.ac.uk

Table of Contents

Supplementary Figure 1: Structure and sequence information for the studied analytes 3

Supplementary Figure 2: Mass spectrum of molecules under investigation at different temperatures denoted on the figure. 4

Supplementary Figure 3: α-synuclein Collision cross section distributions ($^{DT}CCS_{He}$) for $[M+8H]^{8+}$ at seven temperatures spanning 295 K to 190 K 5

Error bars represent the standard deviation from three replicates. Data also shown in Figure 3 of the main text, here with additional of data acquired at 225K 5

Supplementary Figure 4: $^{DT}CCS_{He}^{295K}$ distributions for charge states 7-13+ of α-synuclein at T= 295K from VT-IM-MS. 6

Supplementary Figure 5: Activated Ion Mobility (aIMS) experiments for β-casein............................. 7

Supplementary Figure 6: Selected ATDs and MS obtained during activated ion mobility (aIMS) experiments for β-casein $[M+10H]^{10+}$ 8

Supplementary Figure 7: Activated Ion Mobility (aIMS) data for α-synuclein 9

Supplementary Figure 8: Selected ATDs and mass spectra obtained during activated ion mobility (aIMS) experiments for α-synuclein $[M+8H]^{8+}$ 10

Supplementary Figure 9: Ion activation data of 20μM α-synuclein at different temperatures. 11

Supplementary Figure 10: LHS panel is zoomed mass spectrum of β-casein $[M+10H]^{10+}$, the data shows some sodiated species A, C, D. RHS panel shows the corresponding ATDs for those denoted. 12

Supplementary Figure 11: Hypothetical 1D gas-phase folding free energy surface schematics of (a) α-synuclein and (b) β-Casein in the gas phase. 13

Supplementary Figure 12: Arrhenius plot based for on rate constant for α-synuclein $[M+13H]^{13+}$ ion at T = 295-190 K. 14

Supplementary Table 1: Collision cross sections of G5 Dendrimer (20μM) at 295K, 275K, 250K, 210K, 190K sprayed from aqueous solution. 15

Supplementary Table 2: Collision cross sections of denatured ubiquitin (20μM) at 295K, 275K, 250K, 210K, 190K in water:MeOH=1:1 solution 15

Supplementary Table 3: Collision cross sections of β-casein (20μM) at 295K, 275K, 250K, 210K, 190K in 50mM ammonium acetate, pH 6.8. 16

Supplementary Table 4: Resolution of G5-Dendrimer at 350K-190K. R_{max} is defined as the theoretical maximum resolution of G5-Dendrimer during VT-IM-MS experiments at a given temperature2....... 17

Supplementary Table 5: Resolution of α-synuclein at 350K-190K (Figure 5 main text). 18

Supplementary Table 6: Biophysical information for the compounds investigated 18

Supplementary Table 7: The E/N ratio calculated for the entire dataset, with the value across five sets of drift voltages for three replicates.. 19

Supplementary Methods 19

Supplementary Discussion.. 21

References 23

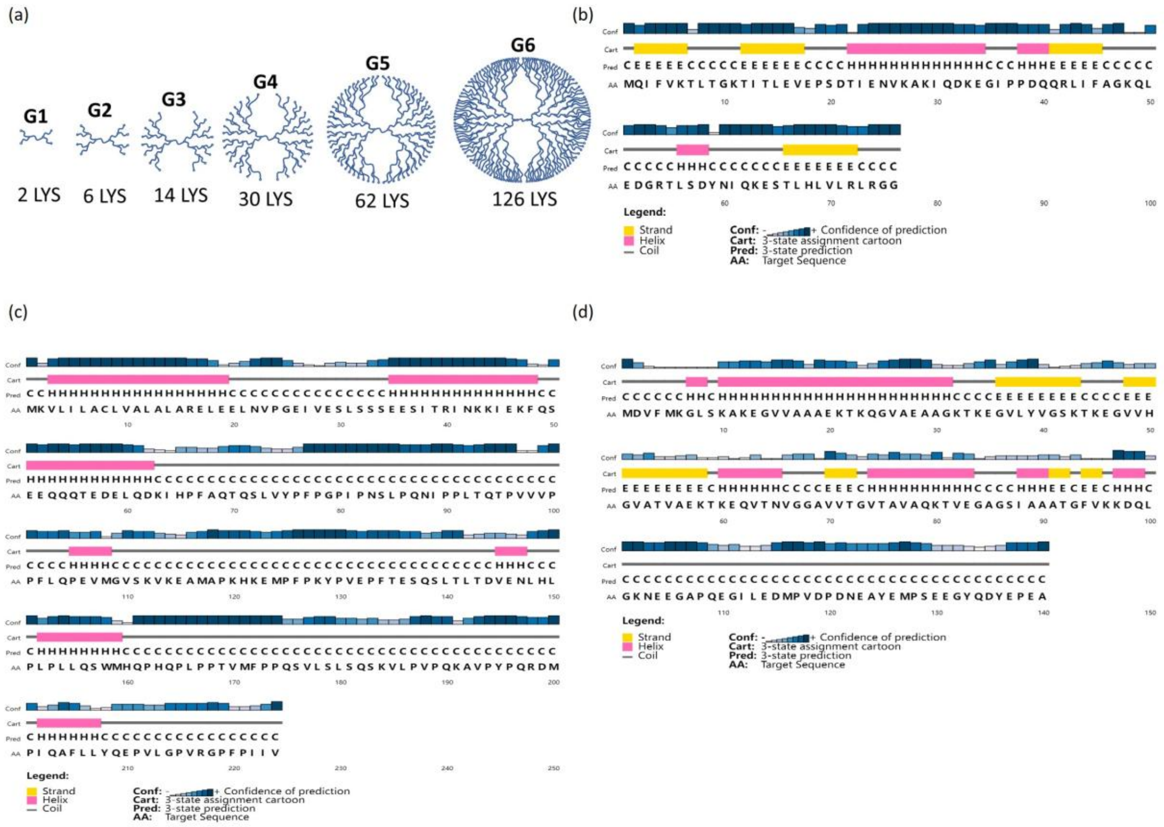

Supplementary Figure 1: Structure and sequence information for the studied analytes:

(a) Schematic of the structures of the G1-G6 PLL dendrimers including the total number of lysine units. (b) amino-acid sequence of Ubiquitin. (c) sequence of β-casein. (d) sequence of α-Synuclein.

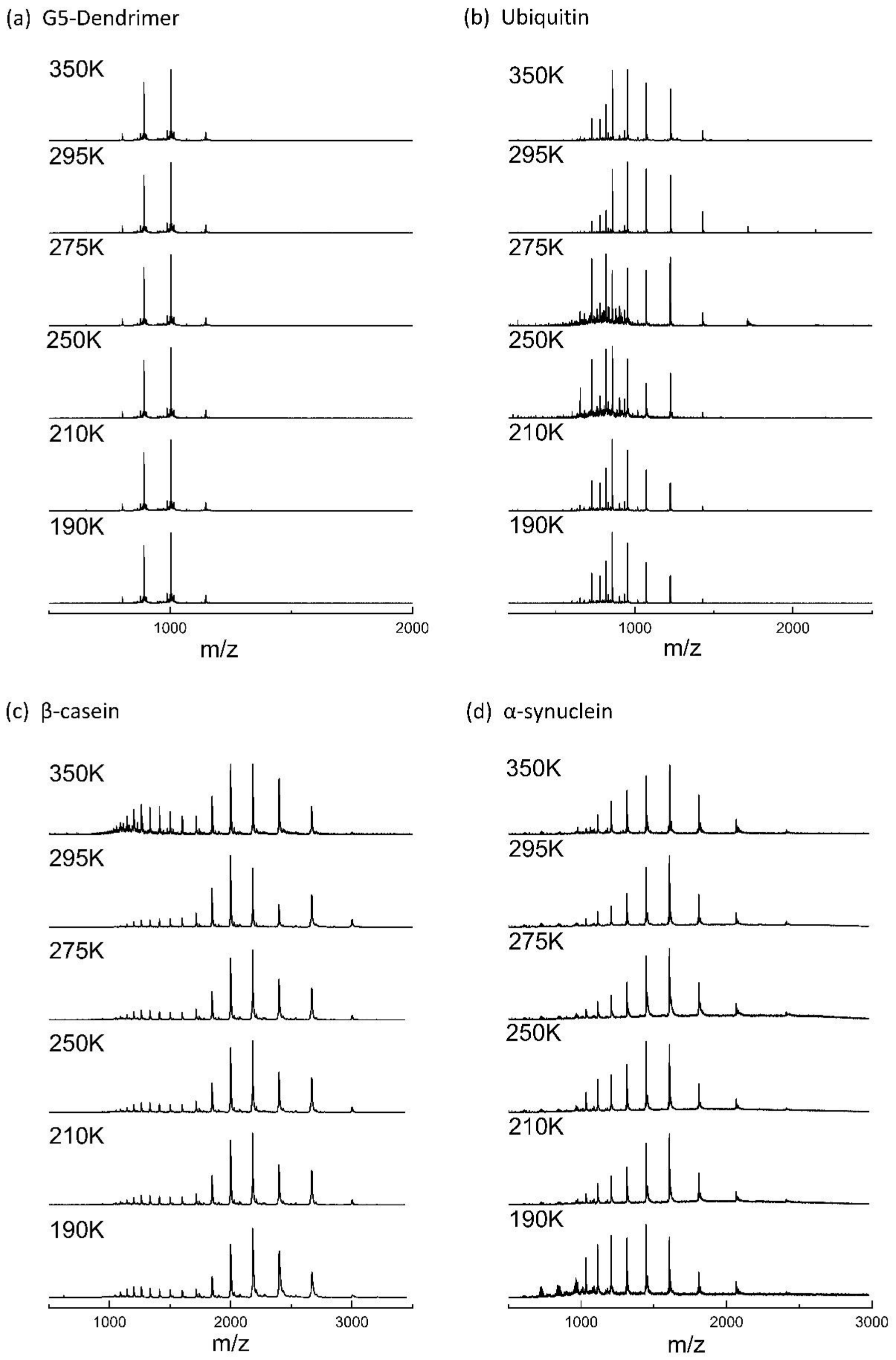

Supplementary Figure 2: Mass spectrum of molecules under investigation at different temperatures denoted on the figure.

a) G5 dendrimer (20µM), b) denatured ubiquitin, c) α-synuclein and d) β-Casein at 295K, 275K, 250K, 210K, 190K in 50mM ammonium acetate, pH 6.8. Data obtained by VT-IM-MS.

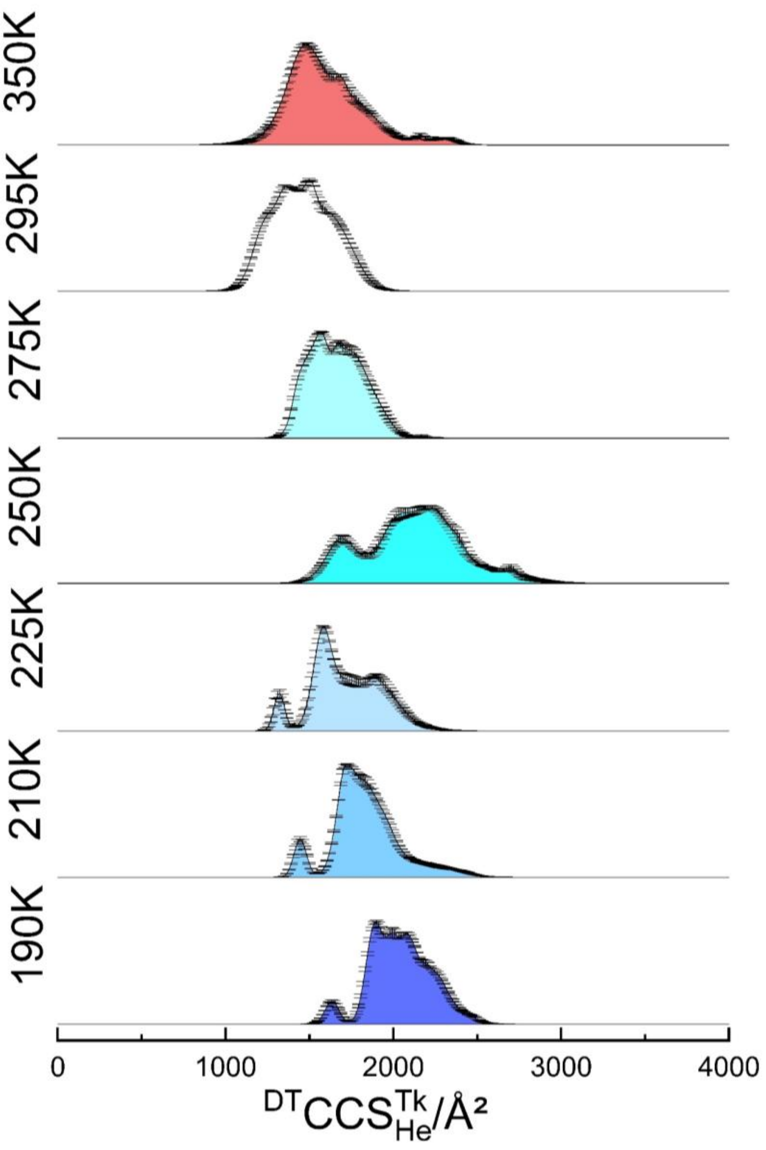

Supplementary Figure 3: α-synuclein Collision cross section distributions ($^{DT}CCS_{He}$) for $[M+8H]^{8+}$ at seven temperatures spanning 295 K to 190 K

Error bars represent the standard deviation from three replicates. Data also shown in Figure 3 of the main text, here with additional of data acquired at 225K.

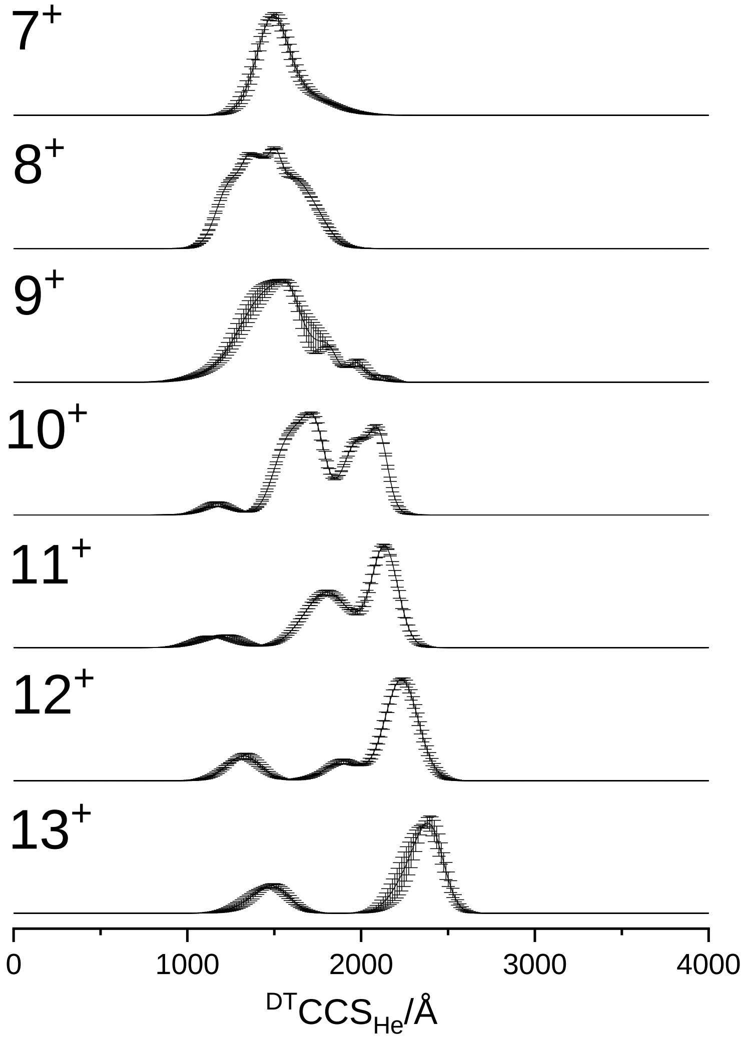

Supplementary Figure 4: $^{DT}CCS_{He}^{295K}$ distributions for charge states 7-13+ of α-synuclein at T= 295K from VT-IM-MS.

nESI spray solution was 20 µM in 50 mM ammonium acetate, with pH 6.8.

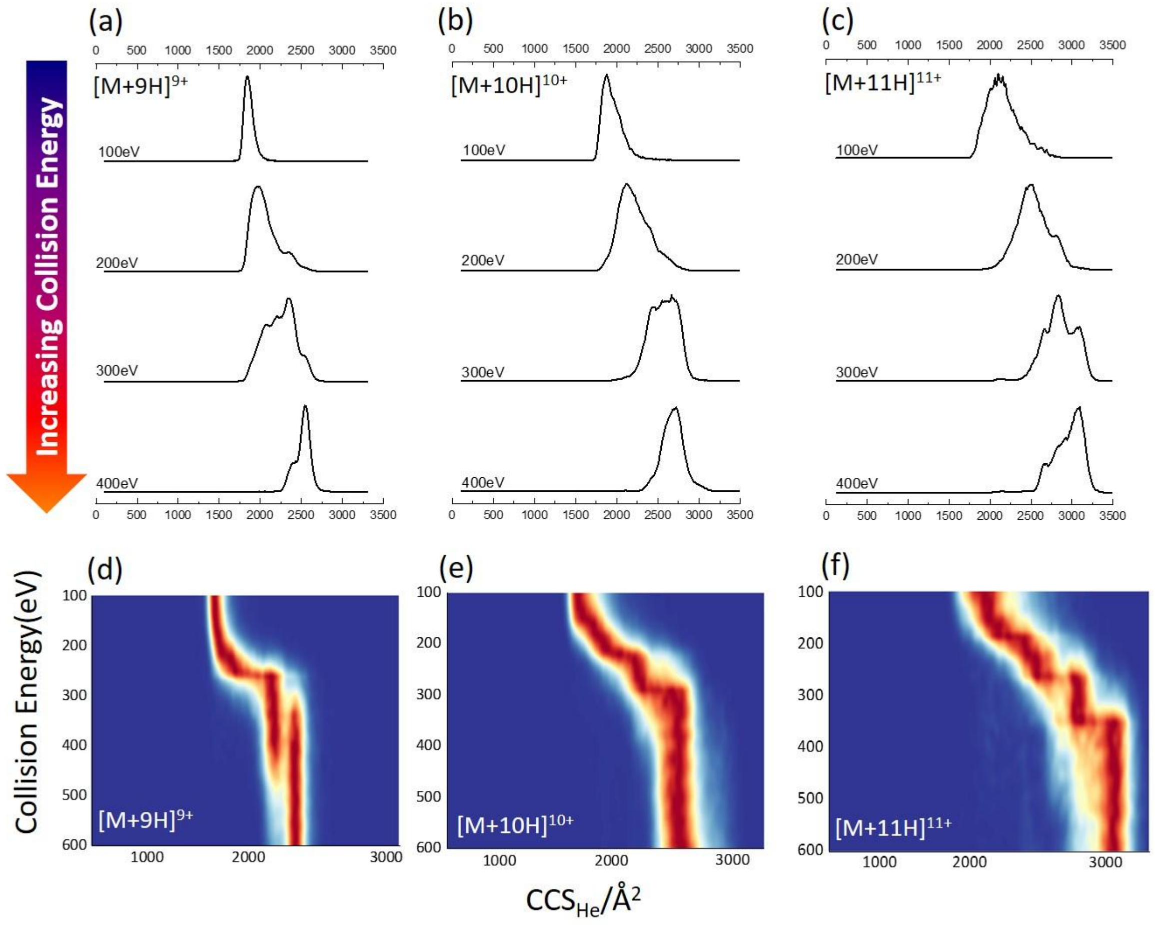

Supplementary Figure 5: Activated Ion Mobility (aIMS) experiments for β-casein

Data shown (a) [M+9H]$^{9+}$ (b) [M+10H]$^{10+}$ and (c) [M+11H]$^{11+}$. Data acquired using Synapt-G2s mass spectrometer with travelling-wave ion mobility. CCS$_{He}$ distribution are calibrated by denatured horse heart myoglobin and cytochrome C. aIMS heat map (d) [M+9H]$^{9+}$, (e) [M+10H]$^{10+}$ and (f) [M+11H]$^{11+}$processed using ORIGAMI[1] software.

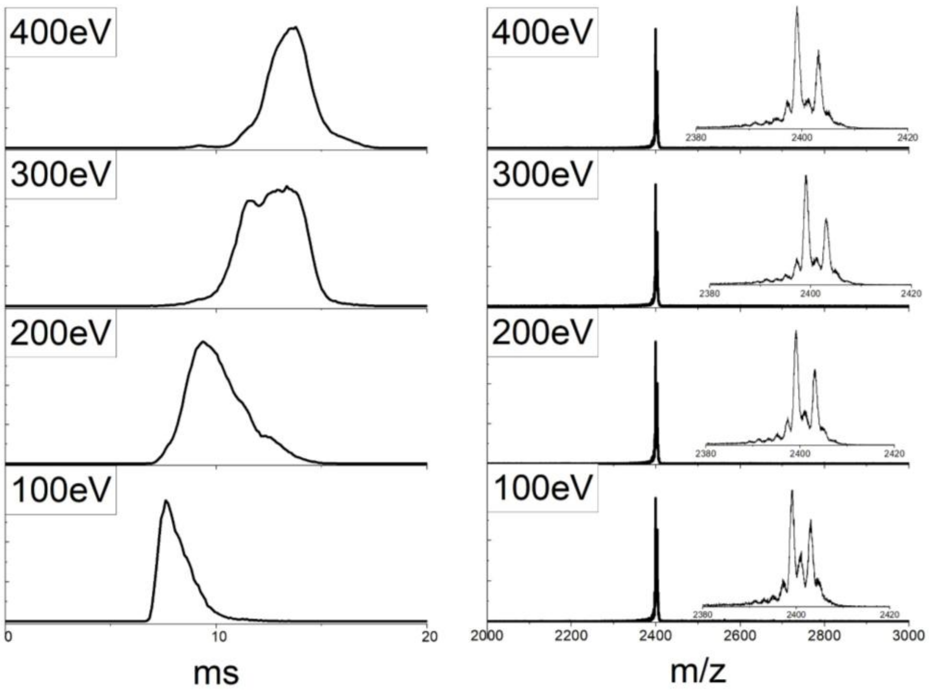

Supplementary Figure 6: Selected ATDs and MS obtained during activated ion mobility (aIMS) experiments for β-casein $[M+10H]^{10+}$

LHS shows the *m/z* selected ATDs and the RHS shows the mass spectrum as a function of collision energy (centre of mass frame) at intervals from 100eV to 400eV. While it is evident that the protein undergoes conformational transitions upon collisional activation, there is no evidence of charge stripping, fragmentation, or dissociation from underlying multimeric species (refer to the zoomed region of the MS on the right-hand side).

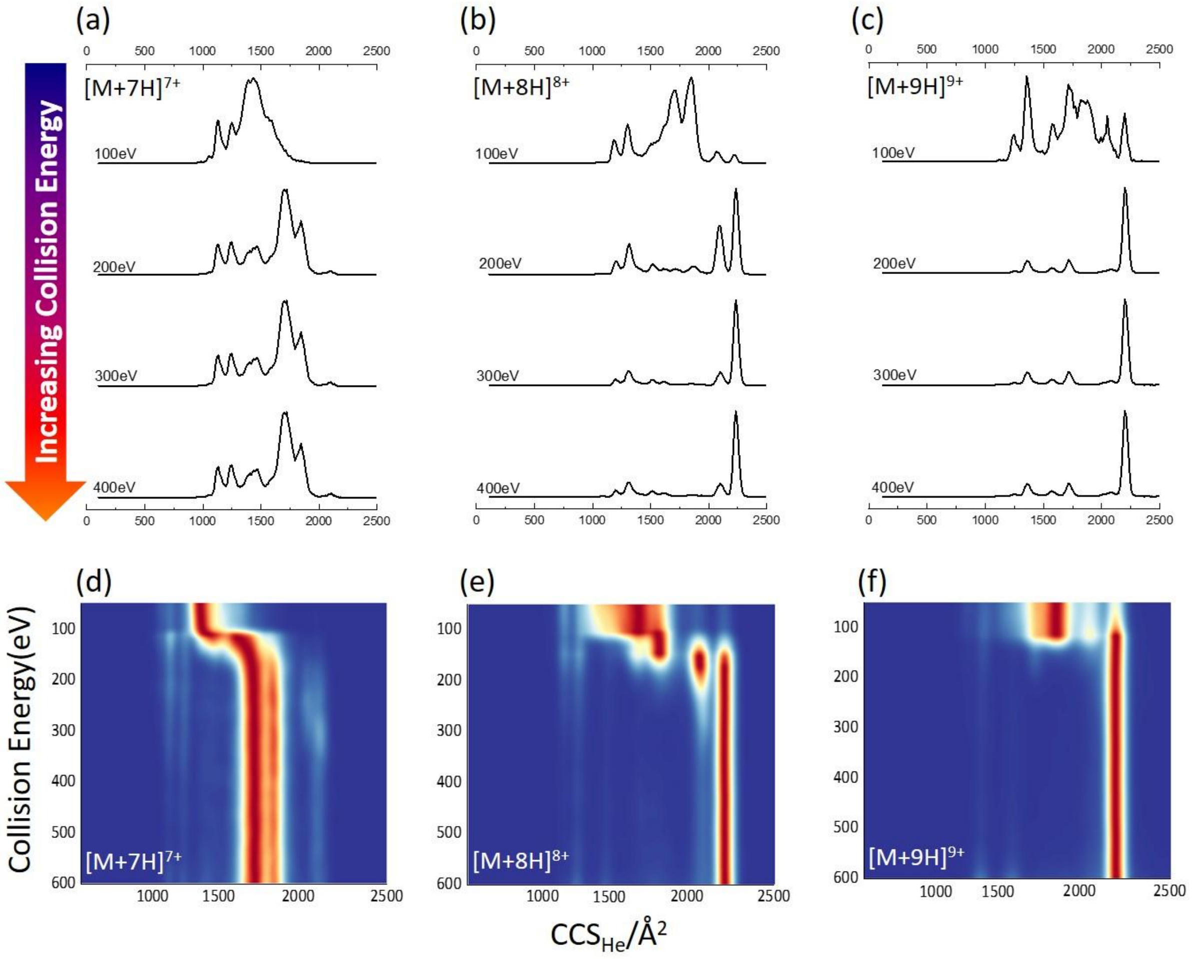

Supplementary Figure 7: Activated Ion Mobility (aIMS) data for α-synuclein

(a) $[M+7H]^{7+}$ (b) $[M+8H]^{8+}$ and (c) $[M+9H]^{9+}$. Data acquired using Synapt-G2s mass spectrometer with travelling-wave ion mobility. CCS_{He} distribution are calibrated by denatured horse heart myoglobin and cytochrome C. aIMS heat maps (d) $[M+7H]^{7+}$, (e) $[M+8H]^{8+}$ and (f) $[M+9H]^{9+}$ processed using ORIGAMI[1] software.

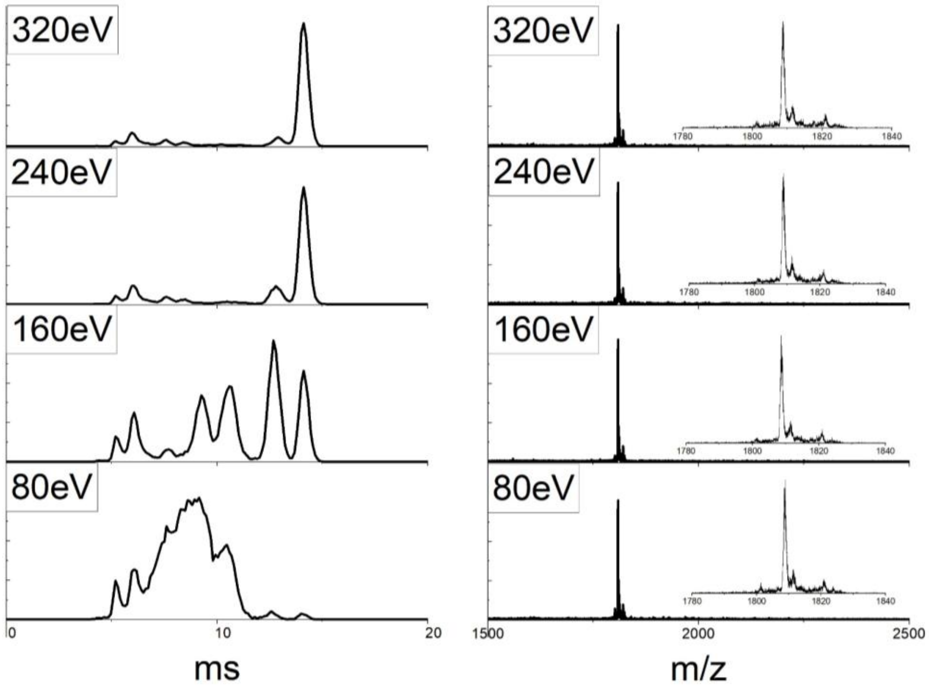

Supplementary Figure 8: Selected ATDs and mass spectra obtained during activated ion mobility (aIMS) experiments for α-synuclein $[M+8H]^{8+}$

LHS shows the *m/z* selected ATDs and the RHS shows the mass spectrum as a function of collision energy (centre of mass frame) at intervals from 100eV to 400eV. While it is evident that the protein undergoes conformational transitions upon collisional activation, there is no evidence of charge stripping, fragmentation, or dissociation from underlying multimeric species (refer to the zoomed region of the MS on the right-hand side).

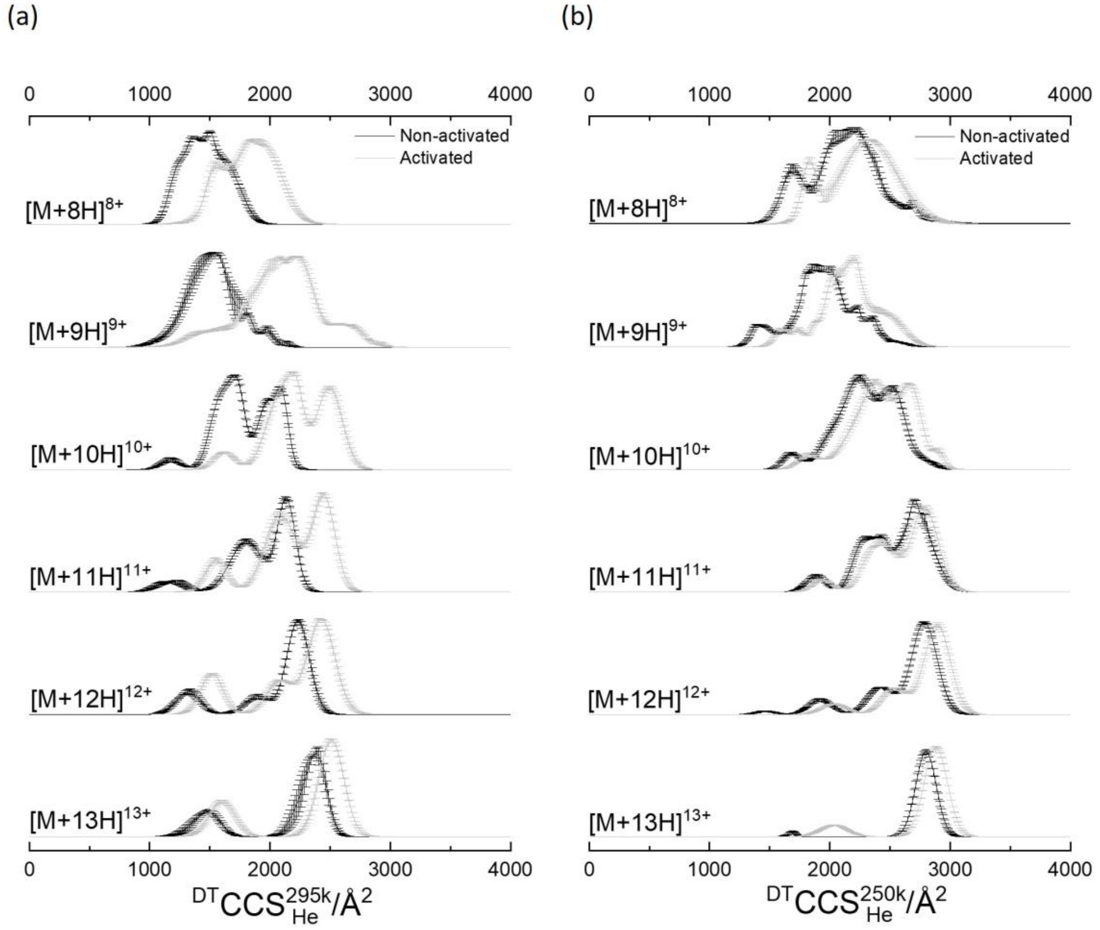

Supplementary Figure 9: Ion activation data of 20μM α-synuclein at different temperatures.

(a) 295K, (b) 250K in 50mM ammonium acetate, pH 6.8. Arranged by VT-IM-MS. Black line represents ATDs of non-activated ions; grey dash line represents activated ion ATDs at an activation voltage of 90V.

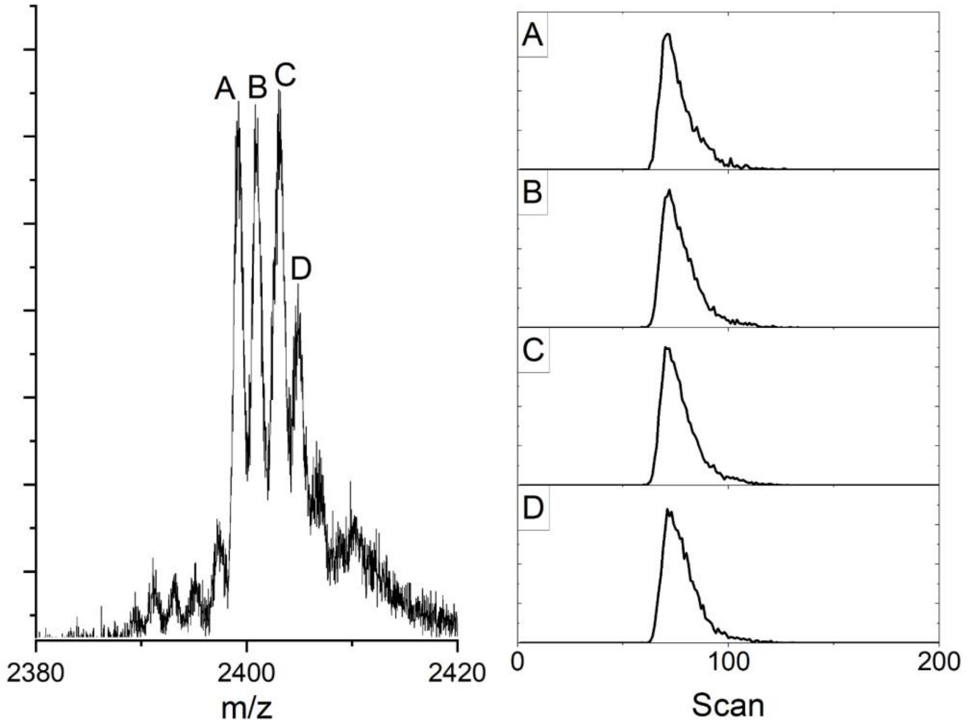

Supplementary Figure 10: LHS panel is zoomed mass spectrum of β-casein $[M+10H]^{10+}$, the data shows some sodiated species A, C, D. RHS panel shows the corresponding ATDs for those denoted.

The similarity of the ATDs (RHS) suggests the conformers adopted are highly similar. The data is from experiments performed on the Synapt G2-Si.

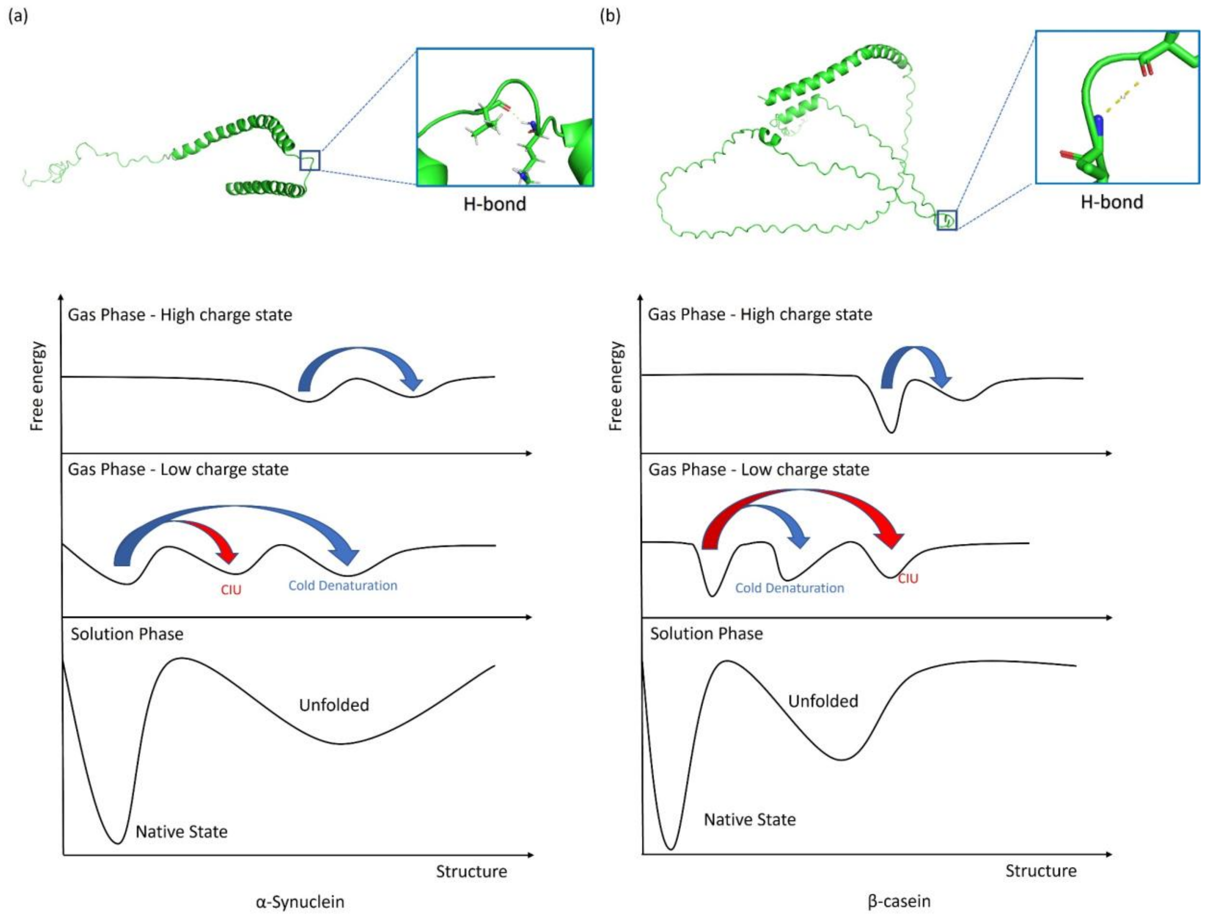

Supplementary Figure 11: Hypothetical 1D gas-phase folding free energy surface schematics of (a) α-synuclein and (b) β-Casein in the gas phase.

For α-synuclein, the cold-denatured conformation at 250K is more extended, for β-Casein, the aIMS-activated conformation is more extended.

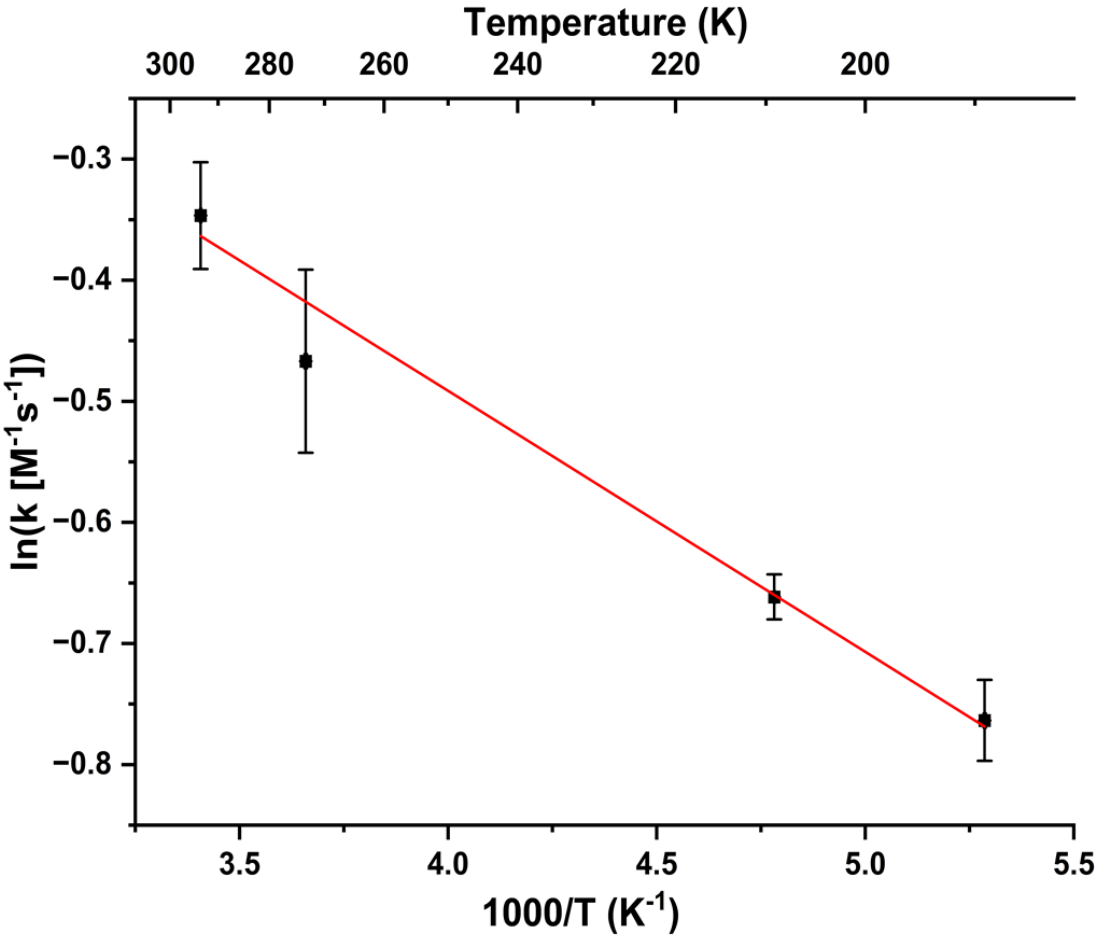

Supplementary Figure 12: Arrhenius plot based for on rate constant for α-synuclein $[M+13H]^{13+}$ ion at T = 295-190 K.

				$^{DT}CCSD_{He}$					ΔCCS			
charge state	m/z	350K	295K	275K	250K	210K	190K	350K	275K	250K	210K	190K
8	1004	1326	1351	1352	1355	1359	1369	-1.85%	0.07%	0.30%	0.59%	1.33%
9	892	1426	1454	1457	1474	1504	1508	-1.93%	0.21%	1.38%	3.44%	3.71%
10	803	1495	1508	1513	1534	1544	1558	-0.86%	0.33%	1.72%	2.39%	3.32%
11	730	1509	1514	1534	1538	1566	1576	-0.33%	1.32%	1.59%	3.43%	4.10%

Supplementary Table 1: Collision cross sections of G5 Dendrimer (20µM) at 295K, 275K, 250K, 210K, 190K sprayed from aqueous solution.

				$^{DT}CCSD_{He}$					ΔCCS			
charge state	m/z	350K	295K	275K	250K	210K	190K	350K	275K	250K	210K	190K
7	1225	1357	1297	1368	1372	1399	1508	4.63%	5.47%	5.78%	7.86%	16.27%
8	1072	1653	1563	1630	1652	1702	1794	5.76%	4.29%	5.69%	8.89%	14.78%
9	954	1686	1626	1661	1673	1712	1824	3.69%	2.15%	2.89%	5.29%	12.18%
10	858	1961	1914	1925	1956	2093	2191	2.46%	0.57%	2.19%	9.35%	14.47%

Supplementary Table 2: Collision cross sections of denatured ubiquitin (20µM) at 295K, 275K, 250K, 210K, 190K in water:MeOH=1:1 solution

				$^{DT}CCSD_{He}$					ΔCCS			
charge state	m/z	350K	295K	275K	250K	210K	190K	350K	275K	250K	210K	190K
9	2665	1911	1864	1877	2062	1916	1924	2.52%	0.60%	10.66%	2.79%	3.23%
10	2400	1920	1895	1938	2050	1985	1990	1.32%	2.27%	8.18%	4.75%	5.01%
11	2181	2001	1923	1934	2071	1939	1976	4.06%	0.55%	7.66%	0.81%	2.71%
12	1999	2151	2147	2159	2259	2192	2249	0.19%	0.54%	5.21%	2.10%	4.75%
13	1846	2344	2326	2403	2448	2427	2433	0.77%	3.29%	5.23%	4.32%	4.59%
14	1714	2594	2579	2618	2712	2623	2686	0.58%	1.51%	5.16%	1.71%	4.15%
15	1600	2707	2693	2715	3151	2771	2856	0.52%	0.82%	17.01%	2.90%	6.05%
16	1500	3385	3371	3376	3890	3550	3671	0.42%	0.15%	15.40%	5.31%	8.90%
17	1412	3624	3604	3729	4006	3832	3853	0.55%	3.47%	11.15%	6.33%	6.91%
18	1333	3942	3903	3945	4277	4020	4112	1.00%	1.08%	9.58%	3.00%	5.35%
19	1263	4016	3971	4076	4319	4140	4209	1.13%	2.64%	8.76%	4.26%	5.99%
20	1200	4209	4157	4241	4476	4303	4311	1.25%	2.02%	7.67%	3.51%	3.70%
21	1143	4376	4351	4369	4442	4376	4408	0.57%	0.41%	2.08%	0.57%	1.31%
22	1091	4580	4559	4596	4752	4605	4631	0.46%	0.81%	4.23%	1.01%	1.58%

Supplementary Table 3: Collision cross sections of β-casein (20μM) at 295K, 275K, 250K, 210K, 190K in 50mM ammonium acetate, pH 6.8.

G5		R_{max}					
charge state	m/z	350K	295K	275K	250K	210K	190K
8	1004	85.4	92.4	97.2	99.0	108.2	114.3
9	892	90.6	98.0	103.1	105.0	114.8	121.3
10	803	95.5	103.3	108.6	110.7	121.0	127.8
11	730	100.2	108.4	113.9	116.1	126.9	134.1
G5		R_{exp}					
charge state	m/z	350K	295K	275K	250K	210K	190K
8	1004	14.6	26.3	17.0	13.6	12.5	27.0
9	892	23.0	10.7	10.8	11.0	12.0	13.0
10	803	21.7	17.3	15.0	12.2	13.6	14.6
11	730	20.3	21.7	28.0	12.2	15.8	17.0
G5		R_{exp}/R_{max}					
charge state	m/z	350K	295K	275K	250K	210K	190K
8	1004	17.1%	28.5%	17.5%	13.7%	11.6%	23.6%
9	892	25.4%	10.9%	10.5%	10.5%	10.5%	10.7%
10	803	22.7%	16.7%	13.8%	11.0%	11.2%	11.4%
11	730	20.3%	20.0%	24.6%	10.5%	12.4%	12.7%

Supplementary Table 4: Resolution of G5-Dendrimer at 350K-190K. R_{max} is defined as the theoretical maximum resolution of G5-Dendrimer during VT-IM-MS experiments at a given temperature2.

α-synuclein	R_{max}						
Charge State	350K	295K	275K	250K	225K	210K	190K
7	79.6	84.7	93.8	91.9	97.1	105.5	105.5
13	108.5	115.4	119.5	125.3	132.3	137.0	143.7
α-synuclein	R_{exp}						
Charge State	350K	295K	275K	250K	225K	210K	190K
7	5.9	9.2	13.8	16.4/18.0/12.2	21.2/ 9.1	11.2	11.5
13	21.2/ 17.2	8.2/ 17.3	27.0/ 25.5	9.7/17.6	23.3/ 17.1	12.7/ 16.4	27.5/ 17.4
α-synuclein	R_{exp}/R_{max}						
Charge State	350K	295K	275K	250K	225K	210K	190K
7	7.4%	10.9%	14.7%	17.9%/19.6%/13.3 %	21.8%/ 9.4%	10.6%	10.9%
13	19.5%/15.9 %	7.1%/ 15.0%	22.6%/ 21.3%	7.8%/14.1%	17.6%/ 13.0%	9.2%/ 12.0%	19.1%/ 12.1%

Supplementary Table 5: Resolution of α-synuclein at 350K-190K (Figure 5 main text).

When more than one conformer is resolved there are more than one value given for R_{exp} given as the conformers resolved from left to right in appearance. R_{max} is defined as the theoretical maximum resolution of α-synuclein on VT-IMMS at a certain temperature[2].

	Predicted Mass (average)	Measured Mass using VT-IMS instrument	Sequence Length	Kappa value
G5-Dendrimer	8020	8020 ± 0.4	-	-
Ubiquitin	8580	8580 ± 0.6	76	0.168
β-casein	23980	23980 ± 0.6	224	0.202
α-Synuclein	14460	14460 ± 0.8	140	0.172

Supplementary Table 6: Biophysical information for the compounds investigated

Predicted molecular mass from sequence, and measured mass, number of amino acids, Kappa value of G5-dendrimer, ubiquitin, α-Synuclein and β-casein, calculated by CIDER[3].

	G5-Dendrimer	Denatured Ubiquitin	β-Casein	α-synuclein
350K	9.49-10.63	9.50-10.47	9.48-10.59	9.56-10.67
295K	7.62-8.84	8.19-9.13	7.50-8.44	7.11-8.35
275K	7.04-7.86	7.32-8.06	7.30-8.07	6.70-7.31
250K	6.05-7.52	6.08-7.46	6.03-7.47	5.85-6.83
210K	5.64-6.31	5.66-6.52	5.66-6.29	5.25-5.93
190K	5.22-5.87	5.22-5.88	5.13-5.81	5.09-5.34

Supplementary Table 7: The E/N ratio calculated for the entire dataset, with the value across five sets of drift voltages for three replicates.

The unit of measurement is Townsend (Td). E is determined from the applied voltage across the cell and N is calculated from the ideal gas law using experimental measurements of the pressure and temperature: $p = Nk_BT$

Supplementary Methods

Activated ion mobility (aIMS)

These experiments (Supplementary Figures 5-8) were performed using a SYNAPT G2-Si Mass Spectrometer as previously reported[1,4]. In this technique, the analyte is subjected to collisions with gas molecules (Argon) in the collision cell of a mass spectrometer, which raises the internal energy of the ions and induces unfolding of proteins.

The resulting unfolded protein ions are then separated by their shape and size using travelling wave IMMS. In the case of the $[M+9H]^{9+}$ ion of β-casein the most abundant conformer had a CCS of 1846 Å^2 when the collision energy is less than 100 eV. As the collision energy is increased to 200 eV the CCSD gradually widens and a small number of extended states around 2300 Å^2 are detected. When energy reached 300 eV, the native population has been greatly reduced, replaced by two distinct extended populations centred at 2353 Å^2 and 2541 Å^2. At very high collision energies (greater than 300 eV), all ions occupy the fully extended state with a collision cross section of 2541 Å^2. Compared with the native state, there was an increase in the CCS of ~37.6%. The CCS of native-like β-casein $[M+10H]^{10+}$ and

$[M+11H]^{11+}$ increase 43.6% and 60.8% respectively when the activation energy increases to 400eV. The unfolding behaviour of β-casein in aIMS experiments is pronounced for low-charge state ions ($[M+9H]^{9+}$ ~$[M+15H]^{15+}$), while for high charge state ions($[M+16H]^{16+}$ ~$[M+22H]^{22+}$), an increase in collision energy does not result in a significant extension of their structure, consistent with the finding that protein ions are more extended at higher charge states due to coulombic unfolding effects[5].

Determination of rate constants for dynamic conformational transitions *in vacuo*

According to Mason *et al.,* the drift time distribution of DT-IMS for a single conformation can be calculated from[6]

$$\Phi(0,x,t) = \frac{sae^{-\alpha t}}{2}\left(v_d + \frac{L}{t_d}\right) \times \left[1 - \exp\left(-\frac{{r_0}^2}{4D_y t}\right)\right] \frac{\exp\left(-\frac{(L-v_d t)^2}{4D_x t}\right)}{\sqrt{4\pi D_x t}} \tag{1}$$

v_d represents the drift velocity, t_d is the drift time of an ion travel through the drift cell with the drift distance L, the density s of ion packet starts the drift from the infinitely thin disc of the diameter r_0, parameter α accounts for the loss rate of ions through collision with the buffer gas. Under low field condition, longitudinal and transverse diffusion coefficients are considered to be equal ($D_y = D_x$)[7].

Figure 6 (main text) shows two conformations for α-synuclein $[M+13H]^{13+}$, labelled A and B, where A at short drift time indicates the helix conformation, while conformation B at long drift time corresponds to the unfolded conformation. Hudgin *et al.* and Poyer *et al.* have demonstrated the determination of rate constants for the structural transition between two conformations using the curve-fitting method, based on the shape of their ATDs.[7,8] In this case, the interconversion from the helix A to the unfolded B (A→B) as the ions travel through the drift cell can be derived as below,

$$\Phi(t) = \frac{A_0 e^{-kt_A}}{\sqrt{2\pi}\,w} e^{-(t-t_A)^2/2w^2} + \frac{B_0}{\sqrt{2\pi}\,w} e^{-(t-t_B)^2/2w^2} + \frac{kA_0}{2\Upsilon} e^{-k(t-\theta)} \left[erf\left(\frac{t_A+\beta-t}{\sqrt{2}\,w}\right) - erf\left(\frac{t_B+\beta-t}{\sqrt{2}\,w}\right)\right] \tag{2}$$

where the first two terms in the equation (2) represent the ATDs of conformation A and B without interconversion during their drift time, while the third term indicates the contribution ATD of A→B;

erf represents the error function; A_0 and B_0 correspond to the initial populations in conformation A and B, respectively; *w* represents the natural diffusional width of the ion packet and is given by

$$w = \frac{1}{2}(t_A + t_B)\sqrt{\frac{2k_BT}{qV}} \qquad (3)$$

Υ is the dimensionless constant, equal to $1\text{-}t_A/t_B$; β and ϑ are time constants and correspond to $\frac{kw^2}{\Upsilon}$ and $\frac{\beta}{2} + t_B$, respectively. *k* is the first-order rate coefficient at which the transition A → B occurs. The rate constant *k* at a specific temperature can thus be acquired by fitting the experimental data at various drift voltages (points in Figure 6b of the main text) with the SI Equation (2) above *via* the Curve Fitter in MATLAB. The solid light line in Figure 6b of the main text is the best fit to the experimental ATD using the model of SI equation (2). Assuming an exponential dependence of the activation energy on the rate coefficient the temperature dependence of rate constants can be analysed with the Arrhenius equation,

$$k = Ae^{-E_A/k_BT} \qquad (4)$$

where *A* is frequency factor, k_B represents the Boltzmann constant, E_A is reaction activation energy, and *T* is the temperature. The activation energy for the transition between the two detected conformations resulting from this method is estimated to be 1.79±0.12 kJ/mol. The Arrhenius plot along with the linear fit is displayed in Supplementary Figure 12.

The csv file from this fit and the corresponding Origin file that was used to fit to our data can be found in supplementary data 1 and 2 respectively.

Supplementary Discussion

The effect of temperature on the protein conformers at 250K leads to an increase in the $^{DT}CCS_{He}$ of around 10%, indicating that the protein is able to access more compact states during IM-MS analysis at cold temperatures, than *via* collisionally induced unfolding at ambient gas temperatures. Such cold

induced restructuring (Figure 4, main text) is less pronounced than performing classical collisional aIMS (Supplementary Figure 5) of β-casein.

α-synuclein undergoes collisionally induced unfolding at lower collisional voltages for the same charge states. The maximally extended state form at a collision voltage of 200 eV. The CCS of $[M+7H]^{7+}$ increases from 1380 $Å^2$ to 1722 $Å^2$, a 24.8% increase relative to the non-activated state, which, in turn is less extended than the state populated by cold denaturation (33.6%). Comparing aIMS data (Supplementary Figure 7) with the VT experiments (Figure 5 main text), it becomes evident that cold denaturation of α-synuclein explores a greater conformational space than aIMS (400eV), accessing more extended states.

Since α-synuclein is more disordered compared with β-casein we observe that cold restructuring is more pronounced in an IDP as its conformational diversity increases. We also performed in-source activation experiments on our linear drift tube mass spectrometer[2] (Supplementary Figure 7) which is induced by increasing the voltage between the source cone and the extractor cone in the Z-spray source, both of which are held close to ambient temperature. For $[M+11H]^{11+}$ to $[M+13H]^{13+}$ of α-synuclein, in-source ion activation with the ion mobility drift gas at 250K, led to ions to have a larger CCS i.e. adopt a more extended conformation (Supplementary Figure 7). Collisional activation in-source allows these IDPs to adopt more extended conformations at low temperatures (most marked at 250K).

References

1. Migas, L. G., France, A. P., Bellina, B. & Barran, P. E. ORIGAMI: A software suite for activated ion mobility mass spectrometry (aIM-MS) applied to multimeric protein assemblies. *International Journal of Mass Spectrometry* **427**, 20–28 (2018).
2. Ujma, J., Giles, K., Morris, M. & Barran, P. E. New High Resolution Ion Mobility Mass Spectrometer Capable of Measurements of Collision Cross Sections from 150 to 520 K. *Anal. Chem.* **88**, 9469–9478 (2016).
3. Holehouse, A. S., Das, R. K., Ahad, J. N., Richardson, M. O. G. & Pappu, R. V. CIDER: Resources to Analyze Sequence-Ensemble Relationships of Intrinsically Disordered Proteins. *Biophys J* **112**, 16–21 (2017).
4. France, A. P., Migas, L. G., Sinclair, E., Bellina, B. & Barran, P. E. Using Collision Cross Section Distributions to Assess the Distribution of Collision Cross Section Values. *Anal. Chem.* **92**, 4340–4348 (2020).
5. Konermann, L., Ahadi, E., Rodriguez, A. D. & Vahidi, S. Unraveling the Mechanism of Electrospray Ionization. *Anal. Chem.* **85**, 2–9 (2013).
6. Mason, E. A. & McDaniel, E. W. Transport properties of ions in gases. *NASA STI/Recon Technical Report A* **89**, 15174 (1988).
7. Hudgins, R. R., Dugourd, P., Tenenbaum, J. M. & Jarrold, M. F. Structural Transitions in Sodium Chloride Nanocrystals. *Phys. Rev. Lett.* **78**, 4213–4216 (1997).
8. Poyer, S. *et al.* Conformational Dynamics in Ion Mobility Data. *Anal. Chem.* **89**, 4230–4237 (2017).
