# Supplementary material for: Conformational landscapes of rigid and flexible molecules explored with variable temperature ion mobility-mass spectrometry
Source: Nat Commun. 2025 May 6;16:4183. doi: 10.1038/s41467-025-59065-x (PMC12052783; doi:10.1038/s41467-025-59065-x)
Supplement: Supplementary file 2 — Description of Additional Supplementary Files [file 41467_2025_59065_MOESM2_ESM.pdf]

Description of Additional Supplementary Files

File Name: Supplementary Data 1

Description: Code and .csv files for the Arrhenius fits
